# Proinsulin-Loaded Nanoparticles Suppress Insulitis and Induce Temporary Diabetes Remission

**DOI:** 10.3390/cells15020174

**Published:** 2026-01-19

**Authors:** Maeva Agapoff, Chloé Dubreil, Emmanuelle Waeckel-Énée, Frédéric Geinguenaud, Valérie Manceau, Julien Diana, Barbara Bertocci, Laurence Motte, Peter van Endert

**Affiliations:** 1 Institut Necker Enfants Malades, Université Paris Cité, INSERM, CNRS, F-75015 Paris, France; maeva.agapoff@yahoo.fr (M.A.); dubreil.chloe@gmail.com (C.D.); emmanuelle.enee@inserm.fr (E.W.-É.); vale.manceau@inserm.fr (V.M.); julien.diana@inserm.fr (J.D.); barbara.bertocci@inserm.fr (B.B.); 2 Université Sorbonne Paris Nord and Université Paris Cité, INSERM, LVTS, F-75018 Paris, France; frederic.geinguenaud@univ-paris13.fr (F.G.); laurence.motte-torcheux@univ-paris13.fr (L.M.); 3 Service Immunologie Biologique, AP-HP, Hôpital Universitaire Necker-Enfants Malades, F-75015 Paris, France

**Keywords:** autoantigen, autoimmune diabetes, immune tolerance, aryl-hydrocarbon receptor, regulatory lymphocyte

## Abstract

**Highlights:**

**What are the main findings?**

- Ultrasmall iron oxide nanoparticles loaded with proinsulin and the aryl hydrocarbon receptor ligand ITE can induce temporary or, rarely, lasting remission of overt autoimmune diabetes in the model of non-obese diabetic mice.
- Nanoparticle treatment induces a rapid attenuation of insulitis with marked depletion of CD8^+^ T cells and dendritic cells.

**What are the implications of the main findings?**

- Short-term induction of B cells producing IL-10 upon treatment with nanoparticles suggests that the emergence of regulatory B cells may contribute to a therapeutic effect.
- Proinsulin/ITE ultrasmall nanoparticles may have potential as immunotherapy when combined with complementary approaches promoting long-term restitution of immune tolerance.

**Abstract:**

Autoimmune type 1 diabetes (T1D) results from the failure of the physiologic regulatory mechanisms that are designed to maintain immune tolerance to pancreatic beta cells. Consequently, the design of strategies to restore tolerance to beta cell antigens is an attractive objective of translational research. We have designed ultrasmall nanoparticles (NPs) loaded with a proinsulin (PI) fusion protein and an agonist for the aryl hydrocarbon receptor (AhR), a transcription factor promoting tolerance induction by different immune cells. We report that a 4 week-treatment with these NPs in non-obese diabetic (NOD) mice starting at disease onset induces temporary and sometimes durable disease remission. Mechanistically, short-term NP treatment induces a rapid depletion of islet infiltrates with a dramatic reduction in the number of CD8^+^ T cells and dendritic cells. This is accompanied by the emergence of B lymphocytes producing IL-10. In the rare mice that undergo durable disease remission, the disappearance of islet infiltrates is associated with the emergence of Foxp3^+^ CD4^+^ regulatory T cells, IFN-γ-producing memory T cells in the spleen, and draining lymph nodes (LNs). We conclude that treatment with these NPs could be of interest in the treatment of recent-onset autoimmune diabetes, but is unlikely to be sufficient for the induction of long-term remission as a stand-alone therapy.

## 1. Introduction

Autoimmune T1D is a public health problem with steadily increasing incidence that results in life-long dependence on insulin injections, a risk of secondary complications, and a reduced life expectancy [1]. Given the evidence of a significant remaining beta cell mass at clinical disease onset in many patients, there is a window of opportunity for strategies to prevent disease or induce remission. However, despite a number of encouraging results obtained both in the mouse model of T1D, the NOD mouse, and in patients, there is still a need for preventive or curative strategies with good efficacy in patients [2].

The initial events triggering the autoimmune process in T1D remain poorly understood. However, it is clear that the disease results from a dysregulation of the islet-specific adaptive immune response in which physiological mechanisms of tolerance are overwhelmed by an auto-aggressive response targeting beta cell antigens [3]. Dysregulation involves autoreactive T cells that expand in both number and in the array of self-antigens that they recognize, then acquire higher avidity and become resistant to the action of regulatory T cells. These normally suppress harmful autoreactivity via regulatory cytokines or cell–cell contact [4,5,6]. Although less studied and understood, B lymphocytes can also play both pathogenic and protective roles in T1D. B cells present beta cell antigens captured through their cell surface receptor to autoreactive T cells, and can produce regulatory cytokines such as IL-10 [7,8,9,10].

Strategies of immunointervention in T1D aim to restore the physiological dominance of adaptive tolerance to beta cells. Ideally, this could be performed by antigen-specific strategies aiming to restore tolerance to specific beta cell antigens. Immune tolerance is then hoped to spread to other antigens through the phenomenon of “infectious tolerance” [11]. As the latter approach may afford better safety profiles, significant effort has been and is spent on developing strategies for delivering beta cell antigens in a tolerogenic manner [12]. Administration of PI, the primary T cell-targeted beta cell antigen, at least in the NOD model of T1D [13], can delay or prevent disease, at least in the NOD model of T1D. However, clinical trials of oral, mucosal, or parenteral PI administration have so far been disappointing. This has prompted the development of approaches enhancing the tolerogenic effect of autoantigen delivery [14,15,16,17]. One such approach is to combine a beta cell antigen with a drug or small molecule with a tolerogenic effect. The efficacy of such approaches can be enhanced by packaging the two molecules in a particle. In this manner, the tolerogenic molecule acts directly and predominantly on antigen-presenting cells, capturing them [18,19]. Ligands for the AhR have previously been used as payloads of NPs and have been shown to induce a tolerogenic phenotype in dendritic cells (DCs) but also in T and B lymphocytes [20,21].

We have previously reported the generation and physicochemical characterization of 9 nm super-paramagnetic iron oxide NPs coated with polyethylene-glycol and loaded with the AhR ligand 2-(1′H-indole-3′-carbonyl)-thiazole-4-carboxylic acid methylester (ITE) [20] and/or a PI-containing fusion protein [22]. In comparison to the NPs that are typically used in similar published studies [20,21], these NPs differ by their small size (9 vs. ≥60 nm), their core material (iron vs. gold), and the nature of their recombinant autoantigen. Our study uses a murine PI 1 fusion protein (P3UmPI), in which the autoantigen is preceded by tandem streptococcal immunoglobulin-binding domains and by ubiquitin (Supplementary Figure S1) [23]. The former allows for fusion protein binding to the Fc portion of immunoglobulin G [24], while the latter may accelerate cytosolic PI degradation upon internalization in antigen-presenting cells. In NOD mice, these NPs accumulated in the inflamed pancreas, a phenomenon absent in non-autoimmune C57BL/6 mice. In vitro, NPs loaded with ITE and P3UmPI were internalized efficiently by bone marrow-derived dendritic cells (BM-DCs) and released both payloads intracellularly. BM-DCs pulsed with NPs loaded with P3UmPI stimulated G9C8 CD8^+^ T cells [25] recognizing the insulin epitope B_15-23_. Moreover, NPs loaded with ITE induced expression of the AhR repressor (*AhRR*), which is induced in response to AhR activation [26].

Here, we studied the effect of NPs loaded with ITE and a PI fusion protein on disease in newly diabetic NOD mice. We find that NPs containing both molecules induce temporary or, rarely, durable disease remission. Contrasting with previous similar work [27,28], the therapeutic effect of our NPs likely involves a massive early depletion of islet infiltrates and possibly an induction of IL-10-producing regulatory B cells (Bregs). The latter phenomenon likely depends on the presence of contaminants, including low amounts of lipopolysaccharide (LPS) associated with autoantigen. We propose that our particles could be of interest as a therapy to clear pancreatic islet infiltrates, which, however, will require a combination with additional therapeutic approaches to restore durable immune tolerance.

## 2. Materials and Methods

### 2.1. Study Design

This study was performed to evaluate the impact of NPs carrying a tolerogenic molecule, ITE, and a T1D autoantigen provided as fusion protein P3UmPI on the blood glucose levels of diabetic NOD mice. Initially, in vitro experiments were performed analyzing NP uptake by DCs and the intracellular release and processing of NP cargo. Then, the effect of NPs on diabetes persistence in NOD mice bred and housed in specific pathogen-free conditions was examined. Testing of glycosuria and glycemia was performed in a blinded fashion. Mice were sacrificed when displaying glycemia ≥ 600 mg/dL. Mice from different breeding pairs were randomly assigned to experimental groups. For diabetes incidence, a minimum of 5 mice was required to obtain statistically significant results using the Log-Rank test. For mechanistic studies, B6 or NOD mice were injected with different batches of NPs. Statistical analysis of the results obtained from a minimum of 3 independent experiments usually gives significant differences using two-way ANOVA.

### 2.2. Fusion Protein Expression and Purification

This study was performed using fusion proteins constructed as described by Kratzer et al. [23]. Briefly, these proteins consist of (N- to C-terminal) three immunoglobulin-binding domains from streptococcal protein G in tandem, human ubiquitin, and the selected antigen, murine PI 1 (mPI) or a fragment of myelin oligodendrocyte protein (mMOG; amino acids 28 to 155). The PI fusion protein was produced using two expression systems: *Escherichia coli* (*E. coli*) and insect cells infected by a recombinant baculovirus. Cloning, production, and purification of the fusion protein produced in bacteria, designated P3UmPI, is described in detail in reference [22] and in the Supplementary Materials. To produce fusion proteins in insect cells, designated *mPI* and *mMOG*, synthetic genes encoding the honeybee melittin signal sequence (MKFLVNVALVFMVVYISYIYA), three domains of streptococcal protein G (WP_142324953.1), murine ubiquitin and PI 1 (NM_008386), or the MOG fragment (NP_0349442), were assembled (Gene Art-Invitrogen) and inserted into the baculovirus transfer vector p1393 vector. The mPI construct was cloned using BamHI-BglII sites, and the mMOG fragment using NheI-BglII sites. Correct sequences were confirmed by sequencing the final constructs. Recombinant baculoviruses were produced by co-transfection of the p1393-based constructs with ProEasy™ baculovirus DNA (AB vector) according to the manufacturer’s protocol. Recombinant viruses were obtained by plaque assays and confirmed by sequencing. High-titer virus supernatants were produced in Sf9 (*Spodoptera frugiperda*) cells, and fusion proteins were purified from the supernatants of virus-infected Hi5 (Trichoplusa ni.) cells, both cultured in serum-free Sf-900™ III SFM medium (Gibco, Grand Island, NY, USA) supplemented with L-Glutamine. Fusion proteins were purified by passing Hi5 supernatants over rabbit immunoglobulin immobilized on Sepharose 4B beads. Eluted proteins were quantified by gel electrophoresis using NuPAGE™ equipment (Invitrogen, Carlsbad, CA, USA) and comparison with a Coomassie-stained BSA standard curve (Supplementary Figure S1). Details on the cell culture and protein purification are given in the Supplementary Materials.

### 2.3. Synthesis and Characterization of NPs

Production, physicochemical characterization, and biodistribution of NPs containing bacterially expressed P3UmPI are described in reference [22]. NPs containing insect cell-expressed proteins were produced and characterized using the same protocols with minor modifications and almost identical loading yields, as detailed in the Supplementary Materials.

### 2.4. Endotoxin Test

Purified fusion proteins and protein-loaded NPs were examined for endotoxin contamination using either the PYROGENT™ Plus Gel Clot LAL Assay (Lonza, Basel, Switzerland) or the LAL Chromogenic Endotoxin Quantitation kit (Pierce, Waltham, MA, USA). LAL reagent was added to an equal volume of the sample, and the formation of a clot was assessed. The sensitivity of the gel clot LAL assay we used was 0.125 EU/mL.

### 2.5. Mice and Treatments

All in vivo experiments were conducted in strict accordance with the recommendations of the European Community (86/609/EEC) and the French legislation (decree no. 87/848) for the use and care of laboratory animals. Female C57BL/6J, NOD, and NOD Rag^−/−^ mice were used, bred, and housed in specific pathogen-free conditions. Starting at 10 weeks old, female NOD mice were blindly checked thrice weekly for glycosuria using Combur-Test^®^ (Roche Diagnostics, Rotkreuz, Switzerland) strips. A positive result was verified by checking glycemia using an Accu-Chek (Roche Diabetes Care, Rotkreuz, Switzerland). Mice with two consecutive readings ≥ 250 mg.dL^−1^ were considered diabetic. Mice were euthanized by cervical dislocation upon reaching a glycemia of 600 mg.dL^−1^. NPs were administered intravenously (i.v.) as a bolus of 200 µmolFe/kg in PBS or 5% glucose solution (Fresenius, Bad Homburg, Germany). To test the effect of NPs on diabetes, newly diabetic female NOD mice received 2 injections of NPs loaded with *E. coli*-expressed protein (PEG-P3UmPI or PEG-ITE-P3UmPI) in 200 µL, or 3 injections of NPs loaded with insect cell-expressed proteins (*mPI*, *ITE-mPI* or *ITE-mMOG*) in 100 mL per week for 4 weeks. For mechanistic experiments, groups of 5 female C57BL/6J or NOD mice aged 10 to 12 weeks were treated with 3 i.v. injections distributed over 10 days (day 1, 5, and 8) and euthanized by cervical dislocation on day 10.

### 2.6. Transfer Experiments

NOD Rag^−/−^ mice (aged 8 to 10 weeks) were i.v. injected with 10 × 10^6^ splenic T cells sorted from diabetic NOD mice, using a biotinylated mouse TCR β chain antibody (Biolegend, San Diego, CA, USA, H57-597) and anti-biotin microbeads (Miltenyi, Bergisch Gladbach, Germany). Additionally, 10 × 10^6^ sorted splenic B cells treated ex vivo for 72 h with complete ITE-P3UmPI NPs or sorted from NOD mice previously treated with NP-PEG or NP-PEG-ITE-P3UmPI in vivo (3 injections over 10 days) were co-transferred (10 × 10^6^). T1D incidence was monitored starting 2 weeks post-transfer.

### 2.7. Preparation of Pancreatic Islets

To analyze islet infiltrates by flow cytometry, pancreata were perfused with a solution of collagenase P (Sigma-Aldrich, St. Louis, MO, USA) in HBSS-1% HEPES (0.75 mg.mL^−1^, Roche), then dissected free from surrounding tissues. Pancreata were digested at 37 °C for 8 min. Digestion was stopped by adding cold HBSS with 10% FCS and 1% EDTA followed by extensive washes. Islets were purified by handpicking in 3 consecutive baths of HBSS-10% FCS supplemented with 1% DNAse 1.

To obtain pancreata for histological examination, harvested organs were fixed overnight in Antigenfix (Diapath, Martinengo, Italy) or 4% paraformaldehyde and washed in 50% ethanol before embedding in paraffin and preparing 4 μm sections mounted onto Superfrost^TM^ slides. Two paraffin sections per mouse were stained in hematoxylin and eosin (HE) for insulitis scoring, and 7 were kept blank for histofluorescence experiments.

### 2.8. Insulitis Scoring

For each pancreas, all islets on 2 HE-stained sections were blindly scored for insulitis using the following classification: 0: normal, 1: minor peri-insulitis, 2: extensive (>50%) peri-insulitis 3: moderate intra-insulitis (less than 50% of mononuclear cells) 4: severe insulitis (more than 50% of mononuclear cells and/or loss of islet architecture).

### 2.9. Lymphoid Organ Cell Preparation

Spleen and pancreatic lymph nodes (PLNs) were harvested and ground through a cell strainer in RPMI or HBSS medium supplemented with 10% FCS. Half of the spleens were digested with collagenase following a published protocol [29] before being passed through the strainer. Splenic red blood cells (RBC) were lysed with RBC Lysis Buffer (Biolegend) and washed with IMDM with 10% FCS. Cells were filtered through a 40 µm filter.

### 2.10. Flow Cytometry

The staining buffer was PBS containing 2% FCS, 0.5% EDTA, and 0.1% sodium azide. Immune cells from PLNs and spleens were split into equal halves for flow cytometric analysis; splenocytes from collagenase-digested spleen halves were used for analyzing myeloid cells. Single-cell suspensions were first stained with LIVE_DEAD™ Fixable Lime (506) Viability kit (Invitrogen, Carlsbad, CA, USA), washed, and incubated for 30 min at 4 °C with anti-CD16/CD32 monoclonal antibody (mAb) to block FcγRII/III receptors (TrueStain FcX™, Biolegend). Then, cells were stained with the mAb listed, for experiments with NPs loaded with the *E. coli*-produced protein, in Supplementary Table S1, and for experiments with NPs loaded with the insect cell-expressed proteins, with 2 mAb panels for myeloid and lymphoid populations, detailed in Supplementary Table S2. Gating strategies for myeloid and lymphoid splenic and LN cells were adopted from reference [30] using the antibodies listed in Supplementary Table S2 and the gating strategies illustrated in Supplementary Figure S2. Fluorescent profiles were acquired on a Fortessa LSRII cytometer (BD Biosciences, San Jose, CA, USA), and data were analyzed with FlowJo v10 software (BD Biosciences). To measure cytokine expression, the cell suspensions were incubated withLPS (1 μg/mL, Sigma-Aldrich Escherichia coli O55:B5) for 5h at 37 °C in the presence of a protein transport inhibitor (eBioscience, San Diego, CA, USA), fixed, and then stained using the intracellular staining kit (Biolegend) for IL-4, IL-10, and IDO. For detection of regulatory T cells, cells were surface-stained for TCR-β and CD4, fixed, and then stained for Foxp3, using the corresponding staining kit (eBioscience). In all experiments, dead cells were excluded using Fixable Viability Dye (eBioscience). Absolute counts were determined using a Malassez counting chamber. Further details, including the antibodies used for flow cytometry, are given in Supplementary Tables S1 and S2.

### 2.11. Fluorescent Immunohistochemistry

Formalin-fixed paraffin-embedded sections were analyzed using multiplexed immunofluorescence staining followed by whole-slide scanning analysis to evaluate the composition of the islet immune infiltrate. After deparaffinization and rehydration through an ethanol gradient, epitope retrieval was performed by boiling sections in 1X AntiFixTM Universal Antigen Retrieval Buffer (Biotum–BTM22030, Fremont, CA, USA) for 20 min. Sections were incubated overnight at 4 °C with Tris-buffered saline (TBS) supplemented with 10% donkey serum (Jackson ImmunoResearch, West Grove, PA, USA) and goat serum (CellSignaling Technology, Danvers, MA, USA) to reduce nonspecific binding of secondary antibodies. Subsequently, sections were incubated with panels of 2 or 3 primary antibodies detailed in Supplementary Table S3, prepared in TBS supplemented with 1% BSA, followed by secondary reagents as detailed in Supplementary Table S3. Sections were finally mounted with Vectashield^®^ (Vector Laboratories, Burlingame, CA, USA), and slides were scanned with Nanozoomer 2.0 HT (Hamamatsu, Japan). Immune cells were blindly counted with NDPview (Hamamatsu, Japan) and reported as function of the islet area.

### 2.12. Ex Vivo B Cell Treatment

B cells were magnetically isolated using MojoSort™ Mouse Pan B cell Isolation Kit (Biolegend). The resulting B cells (6 × 10^5^ cells/well in Supplementary Figure S4A–C, 6 × 10^5^/well in Supplementary Figure S3A,B) from 11 to 15-week-old female (non-diabetic) NOD or C57BL/6J mice were incubated for various periods in complete IMDM with 90 μmol or titrated Fe equivalent amounts of NPs, PI fusion protein or vehicle, as indicated. RPMI 1640 medium with 10% FCS was used for the experiments in Supplementary Figure S3A,B. For cytokine detection, cells were incubated with LPS and in the presence of a protein transport inhibitor (eBioscience) for the last 5 h.

### 2.13. Statistical Analysis

Statistical analyses were carried out using GraphPad Prism v10 software. Diabetes incidence was plotted according to the Kaplan–Meier method. The statistical difference between the survival curves was evaluated using a Log-rank (Mantel–Cox) test. The statistical difference between the groups for insulitis scoring was evaluated using a chi-square test for contingency. The statistical differences between the groups for the histofluorescence and flow cytometry experiments were evaluated with one-way or two-way ANOVA, as indicated. A *p*-value of less than 0.05 (* *p* < 0.05, ** *p* < 0.01, and *** *p* <0.001) was considered statistically significant.

## 3. Results

### 3.1. Production of Autoantigen Fusion Proteins

During this study, we first produced PI in a procaryotic expression system, as described in [22], but then performed additional experiments with PI and a control neuronal self-antigen, myelin oligodendrocyte glycoprotein (MOG), produced in a eukaryotic expression system (insect cells; Supplementary Figure S1A–C). The use of these two different systems for PI expression was designed to reveal the potential effects of contaminants, including LPS, that are frequently associated with proteins expressed in *Escherichia coli*. Here we will use the term **P3UmPI** to designate PI produced in *E. coli*, and the terms *mPI* and *mMOG* to designate fusion proteins expressed in insect cells. Results obtained with *E. coli* vs. insect cell-produced proteins will be displayed throughout the manuscript using distinct graphical codes.

### 3.2. NP Treatment of Newly Diabetic NOD Mice

**Figure 1:**
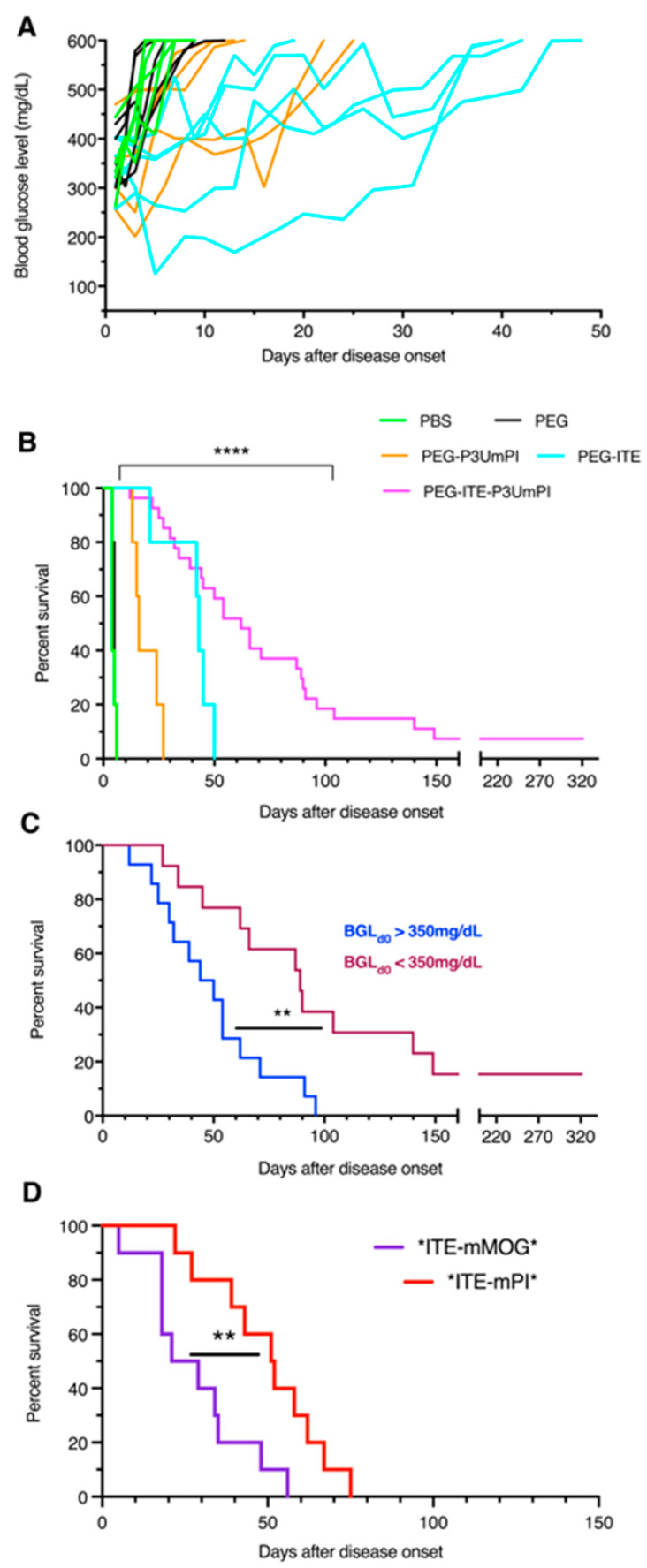
In vivo effect of NPs on diabetes development: (**A**) Starting at diabetes onset, defined as a blood glucose level > 250 mg/dL, groups of five female NOD mice were injected twice weekly i.v. with vehicle (PBS) or NPs loaded with PEG, PEG-ITE, or PEG-P3UmPI, and glycemia was monitored; the glycemia of individual mice is shown. (**B**) Twenty-seven newly diabetic mice were treated with NPs loaded with ITE and P3UmPI, and the effect on survival was compared to the treatments shown in (**A**); N = 27 for PEG-ITE-P3UmPI, N = 5 for all other groups; (**C**) compares the results obtained for diabetic mice injected with (*E. coli*-expressed) ITE-P3UmPI NPs presenting with a glycemia above (N = 14) or below (N = 13) 350 mg/dL at diabetes onset; (**D**) Groups of ten female NOD mice were injected thrice weekly with NPs loaded with (insect cell-expressed) *ITE-mMOG* or *ITE-mPI*, starting at diabetes onset. In all experiments, mice were sacrificed when displaying glycemia ≥ 600 mg/dL. Incidences were compared with the log-rank test. **, *p* < 0.01, ****, *p* < 0.0001.

Experimental immunotherapy of T1D can be applied both during early or late pre-diabetes and at the onset of fasting hyperglycemia, i.e., manifest disease. Considering that treatments that are effective at the onset of clinically manifest disease will likely benefit a greater number of patients, we focused on the latter setting. Female NOD mice were monitored twice weekly for hyperglycemia. Mice displaying glycemia > 250 mg/dL at two consecutive measurements were treated with two weekly intravenous injections of NPs for four weeks. While mice injected with PBS or with PEG-coated NPs all progressed to hyperglycemia > 600 mg/dL within less than ten days, treatment with P3UmPI-loaded NPs slightly delayed this by up to 25 days, and treatment with ITE-NPs by up to 45 days (Figure 1A). In contrast, treatment with NPs loaded with both ITE and P3UmPI delayed terminal hyperglycemia by up to 150 days and induced complete and lasting remission in two mice (Figure 1B). In total, 50% of the mice in this group were alive at 55 days, and 33% at 85 days after onset. Mice presenting with hyperglycemia below 350 mg/dL at the start of the treatment were most likely to benefit from treatment with ITE/P3UmPI NPs, with 50% alive three months after disease onset, vs. 10% for mice with hyperglycemia > 350 mg/dL at the start of treatment (Figure 1C).

We also treated additional groups of ten mice with NPs loaded with insect cell-expressed PI. Here we compared the effect of the PI fusion protein (*mPI*) to the control fusion protein containing myelin oligodendrocyte (*mMOG*), a neuronal self-antigen recognized by autoreactive T cells in the murine model of experimental autoimmune encephalomyelitis [31]. Treatment of mice with *ITE-mMOG* NPs prolonged survival up to 60 days (Figure 1D), comparable to NPs carrying ITE alone (Figure 1B). NPs loaded with *ITE-mPI* prolonged survival significantly longer than *ITE-mMOG* NPs, although less than ITE-P3UmPI NPs. This discrepancy suggested that the method of PI fusion protein production in a bacterial versus eukaryotic (insect cell) system may underlie the variable efficacy of the NP treatment. Thus, PI and ITE prolonged survival in both independent and additive manners, while the control fusion protein had no discernible effect on survival.

### 3.3. Effect of Short-Term NP Treatment In Vivo and Ex Vivo on Splenocytes

**Figure 2:**
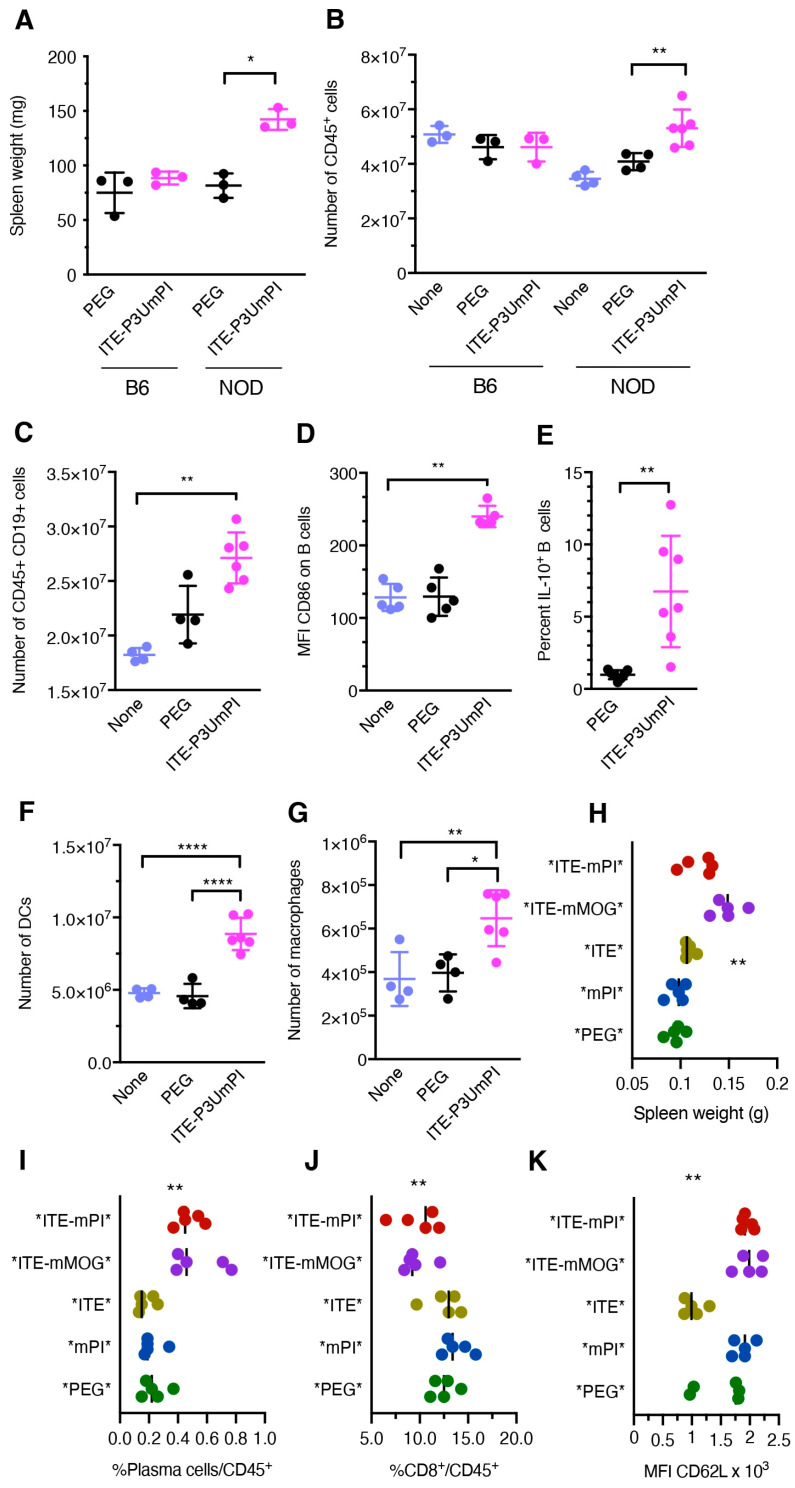
Short-term effect on splenocytes of treatment with NPs carrying *E. coli* or insect cell-expressed proteins. Prediabetic female NOD (**A**–**G**) and control C57BL/6 mice (**A**,**B** only) aged 10–12 weeks were left untreated or treated with three injections of PEG-coated or ITE-P3UmPI-loaded NPs for ten days. Panels (**A**,**B**) compare the weight of the spleens and the total number of CD45^+^ cells. Flow cytometric data in panels (**C**–**G**) indicate the total number of B cells defined as CD45^+^TCR-b^−^CD19^+^ (**C**), CD86 levels on B cells (**D**), IL-10 expression by B cells (**E**), and the total numbers of DCs (**F**) and macrophages (**G**). Panels (**H**–**K**) show flow cytometric data for NOD mice treated with three injections of NPs loaded with insect cell-expressed proteins and indicate spleen weight (**H**), the percentage of CD138^+^ TACI^+^ plasma cells (**I**) and of CD8^+^ T cells (**J**) among hematopoietic splenocytes, and the mean fluorescence intensity (MFI) of CD62L on T cells (**K**). N = 3 to 7 per group from three independent experiments for (**A**–**G**) and five per group for panels (**H**–**K**). Group mean +/− SE values were compared using the two-way ANOVA test for (**A**–**G**) and one-way Anova for (**H**–**K**). *, *p* < 0.05, **, *p* < 0.01, ****, *p* < 0.0001.

Seeking to identify the mechanisms delaying disease progression or inducing remission in diabetic NOD mice, we injected groups of pre-diabetic mice three times with NPs over ten days. We then used flow cytometry (Supplementary Figure S2) to compare the numbers of immune cell subsets and their phenotype in organs of interest to those in untreated or control-treated NOD mice. This was performed with NPs loaded with *E. coli* and insect cell-expressed fusion proteins in independent experiments. First, we examined the spleens, a major site of NP accumulation [22], of mice treated with ITE-P3UmPI NPs. Weighing spleens gave the first indication of the strong effect of these NPs on the splenocytes of pre-diabetic mice treated for ten days. NP treatment almost doubled the spleen weight compared to PEG-coated control NPs, but did not produce such an effect in control C57BL/6 mice (Figure 2A). This was associated with a 15% increase in the number of CD45^+^ cells in NOD mice treated with ITE-3UmPI NPs relative to PEG-NP-treated mice, while both complete (ITE + P3UmPI) and control NPs were without effect in C57BL/6 mice (Figure 2B). Analysis of lymphocyte populations revealed a strong increase in the number of B cells in the enlarged spleens (Figure 2C). These B cells were activated, as documented by CD86 upregulation (Figure 2D), and significant proportions of them produced IL-10, IL-4, and indoleamine dioxygenase (Figure 2E, Supplementary Figure S3A,B). Consistent with a strong humoral response to ITE-P3UmPI NPs, treated mice also harbored increased serum levels of IgM and IgG (Supplementary Figure S3C,D). Enlarged spleens contained increased numbers of DCs and macrophages (Figure 2F,G), while CD86 expression by these populations was not altered (Supplementary Figure S3E,F). The number of T cells and the percentage of CD4^+^Foxp3^+^ regulatory T cells did not differ between mice treated with control PEG NPs and ITE-P3UmPI NPs (Supplementary Figure S3G,H).

A repeat experiment with NPs loaded with insect cell-expressed proteins confirmed the increased spleen weight; however, this was observed for both fusion proteins in combination with ITE (Figure 2H). Although the total number of B cells was not increased, both fusion proteins induced an increase in plasma cells, but once again, only when associated with ITE (Figure 2I). Moreover, both induced a reduction in the number of CD8^+^ T cells (Figure 2J) and higher expression of CD62L on T lymphocytes compared with NPs loaded only with ITE (Figure 2K). 

Collectively, these results indicated that, in the spleen, short-term treatment with NPs loaded with fusion proteins and ITE induced the expansion of immune cells; however, these had different focuses: activated B cells producing IL-10 and myeloid cells for *E. coli*-produced PI, versus plasma cells for insect cell-expressed PI. However, the effects shared between *mPI* and *mMOG*-loaded NPs suggest that the common moieties of the fusion proteins, most likely the streptococcal protein G domains, in conjunction with ITE, may underlie some of the effects observed. In addition, the stronger effects of ITE-P3UmPI NPs compared to *ITE-mPI* NPs, consistent with stronger induction of a humoral response, suggested that the source of the fusion proteins, e.g., potential contaminants, could have contributed to the biological effects.

We considered that IL-10 production by B lymphocytes could be induced by signaling through Toll-like receptors, including TLR4 bound by LPS. This, in turn, can result in the production of regulatory B lymphocytes, stimulating the emergence of regulatory Tr1 cells [32,33]. We examined the LPS content and IL-10 stimulatory capacity of bacterial and insect cell-expressed proteins. Incubation of purified splenic B lymphocytes induced dose-dependent secretion of IL-10 that was about 2- to 3-fold higher for P3UmPI-NPs than for NPs loaded with either protein produced in insect cells (Supplementary Figure S4A,B). However, relatively large amounts of NPs (exceeding one dose injected in vivo) loaded with each of the three fusion proteins were significantly less potent than a low amount (5 mg/mL) of purified LPS regarding IL-10 induction (Supplementary Figure S4B). Direct measurement of endotoxin content indicated a range of 0.5 to 4 endotoxin units per mL for proteins produced in the bacterial and eukaryotic systems and NPs loaded with them (Supplementary Figure S4C). Assuming a conversion of about 0.2 ng endotoxin per EU and considering the established minimal concentration of 100 ng LPS for IL-10 production by B lymphocytes [31], low-level endotoxin contamination was unlikely to account alone for stronger IL-10 production and diabetes protection by P3UmPI NPs. This suggested a role for other undefined contaminants.

We wished to further characterize the strong effect of ITE-P3UmPI NPs on B cells and determine whether this was a direct effect of NPs or required the presence of other populations. We sorted splenic B cells from prediabetic NOD and C57BL/6 mice, incubated them for three days with NPs, and analyzed their phenotype and effector functions. NPs loaded with ITE and P3UmPI alone had opposite effects on B cell activation, with an increase for P3UmPI that was abolished by a dominant decrease mediated by ITE (Supplementary Figure S5A). Both NPs carrying P3UmPI alone and ITE-P3UmPI NPs induced the production of the regulatory cytokine IL-10. C57BL/6 B cells responded more strongly to P3UmPI in the absence of ITE (Supplementary Figure S5B). In contrast, only NOD splenic B cells responded to NPs loaded with P3UmPI (alone or in combination with ITE) with production of TGF-b (Supplementary Figure S5C).

We wondered whether B cells producing IL-10 and/or TGF-β generated ex vivo or in vivo were able to delay or prevent T1D. To address this, we adoptively transferred diabetogenic splenic T cells obtained from recently diabetic NOD mice, together with sorted B cells from the spleen of NP-treated prediabetic NOD mice, or with B cells incubated with ITE-P3UmPI NPs ex vivo, to immunodeficient NOD Rag^−/−^ mice and monitored diabetes occurrence. Co-transfer of B cells from mice treated with PEG NPs did not delay diabetes appearance due to diabetogenic T cells; all mice were diabetic by 45 days after transfer. However, B cells treated ex vivo with ITE-P3UmPI NPs, or obtained from mice treated with the same NPs, delayed disease; in these groups, 100% diabetes was reached only on day 80 or 75, respectively (Supplementary Figure S5D). B cells treated ex vivo conferred the strongest protection, with a mean delay of 61 days vs. 49 days for B cells treated with complete NPs in vivo. Mice receiving diabetogenic T cells only, or T and B cells from control PEG-NP-treated mice, developed diabetes on average after 33 and 37 days, respectively. We concluded that short-term ex vivo treatment of splenic B cells with ITE-P3UmPI NPs induces the expansion of B cells producing regulatory cytokines that may be capable of inhibiting disease transfer by diabetogenic T cells.

### 3.4. Effect of Short-Term NP Treatment on Immune Cells in PLNss

**Figure 3:**
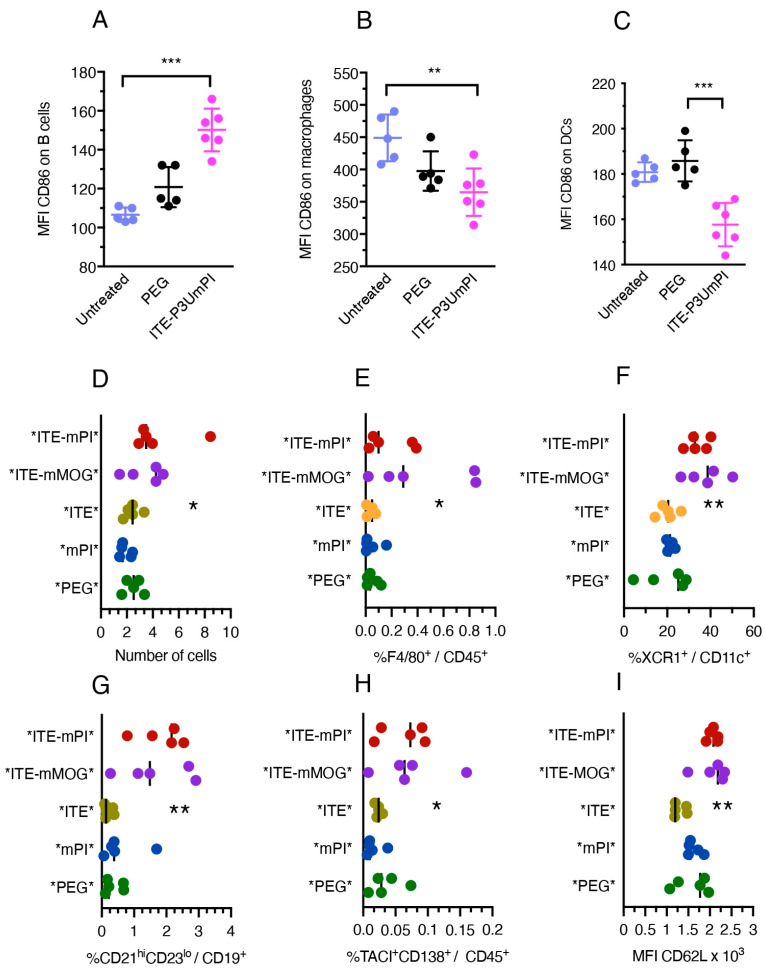
Short-term effect of NP treatment on PLN cells. Groups of five or six mice were injected with control NPs or NPs loaded with fusion proteins + ITE, as indicated, for ten days before flow cytometric analysis of immune cells. Panels (**A**–**C**) show the effect of ITE-P3UmPI NPs on CD86 expression by PLN B lymphocytes (**A**), macrophages (**B**), and DCs (**C**). Panels (**D**–**I**) show the total number of PLN cells (**D**), the number of macrophages (**E**), and the percentages of type 1 conventional DCs among total DCs (**F**); marginal zone-like cells among B cells (**G**); plasma cells among immune cells (**H**); and the MFI of CD62L staining on T cells (**I**). Values for individual animals and group means are shown and were compared by one-way ANOVA (Friedman test); panels (**A**–**C**) also show standard errors. *, *p* < 0.05, **, *p* < 0.01, ***, *p* < 0.001.

Next, we analyzed the effect of short-term treatment with NPs on immune cells inPLNs. Like in the spleen, ITE-P3UmPI NPs induced upregulation of CD86 by B cells, surprisingly contrasting with CD86 downregulation on LN macrophages and DCs (Figure 3A–C). Treatment with NPs loaded with *mPI* or *mMOG* and ITE produced effects corresponding to a collection of the NP effects observed for splenocytes. This included an increase in the total number of immune cells and in the number of macrophages and cDC1 (Figure 3D–F) as well as increases in the percentages of marginal zone-like B cells and of plasma cells (Figure 3G,H). CD62L expression was lower on LN T cells from mice receiving single-loaded or “empty” NPs than in mice injected with double-loaded NPs (Figure 3I). Again, the effects of NPs loaded with ITE plus *mPI* or *mMOG* were essentially identical, suggesting a synergistic effect of common moieties of the fusion proteins with ITE. Thus, in both the spleen and the PLNs, NPs loaded with ITE plus fusion proteins (expressed in *E. coli* or insect cells) induced an immune response with increased cellularity and amplification of global B cell numbers or plasma cells, as well as myeloid cells. However, as was observed for both fusion proteins, this response may have been triggered by shared fusion protein moieties or contaminants rather than the autoantigens. Moreover, fusion-protein-loaded NPs could induce production of regulatory cytokines by splenocytes, although this effect was independent of the autoimmune setting in the NOD strain and potentially limited to *E. coli*-produced fusion protein.

### 3.5. Effect of Short-Term NP Treatment on Islet-Infiltrating Immune Cells

**Figure 4:**
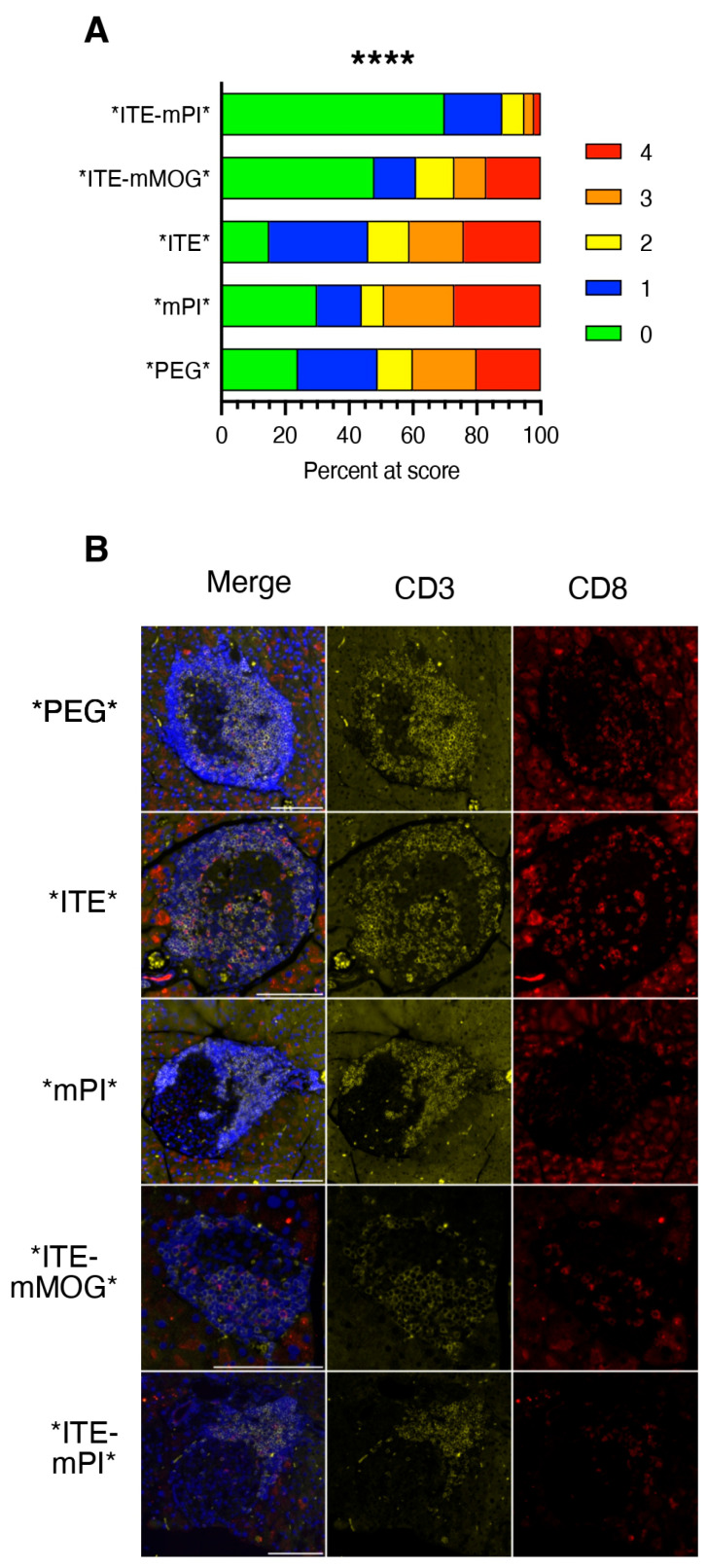
Short-term effect of NP treatment on islet infiltration. Groups of five female NOD mice aged 10–12 weeks were subjected to a ten-day treatment with the NPs indicated and islet infiltration was scored blindly by examination of hematoxylin-eosin-stained sections (two sections per mouse) (**A**), using the following scale: 0 = no infiltrate, 1 = peri-islet infiltrate, 2 = extensive (>50%) peri-islet infiltrate, 3 = intra-islet infiltrate, and 4 = extensive/destructive intra-islet infiltrate (>50%). The total number of islets per group ranged between 117 (*PEG*) and 241 (*ITE-mPI*). Results were evaluated by Chi-square test. ****, *p* < 0.0001. (**B**) Example of immunofluorescent staining of pancreas sections from short-term NP treated mice for quantification of islet-infiltrating immune cells. FFPE sections of islets from mice treated with the NPs indicated at the left were stained with antibodies to CD3 and CD8 and secondary reagents coupled to Alexa Fluor 594 (CD3) or 647 (CD8). T cell staining reveals peri-insulitis and intra-insulitis of various intensities in all sections except sections from *ITE-mPI*NP-treated mice that only show peri-insulitis. Note the strong autofluorescence of exocrine tissue at 647 nm. Scale bar = 100 mm.

Next, we subjected pre-diabetic female NOD mice to treatment with the different NPs and evaluated their effects on islet-infiltrating immune cells using classic histology, flow cytometry, and fluorescent immunohistochemistry. Figure 4A shows the result of the blinded scoring of hematoxylin-eosin-stained pancreas sections from groups of five mice treated with the NPs indicated for ten days. Importantly, islets from *ITE-mMOG*-treated mice displayed a percentage of grade 3 or 4 infiltrated islets, like islets from mice treated with “empty” or single-loaded NPs. In contrast, islets from mice treated with *ITE-mPI* NPs showed a dramatic decrease in grade 3 or 4 infiltrated islets. Thus, contrasting with the shared effects of the two NPs carrying a fusion protein plus ITE in PLNs, only NPs loaded with the islet autoantigen PI suppressed infiltration. This is illustrated in Figure 4B, showing the scarcity of CD3^+^ and particularly CD8^+^ cells in islets from this group.

**Figure 5:**
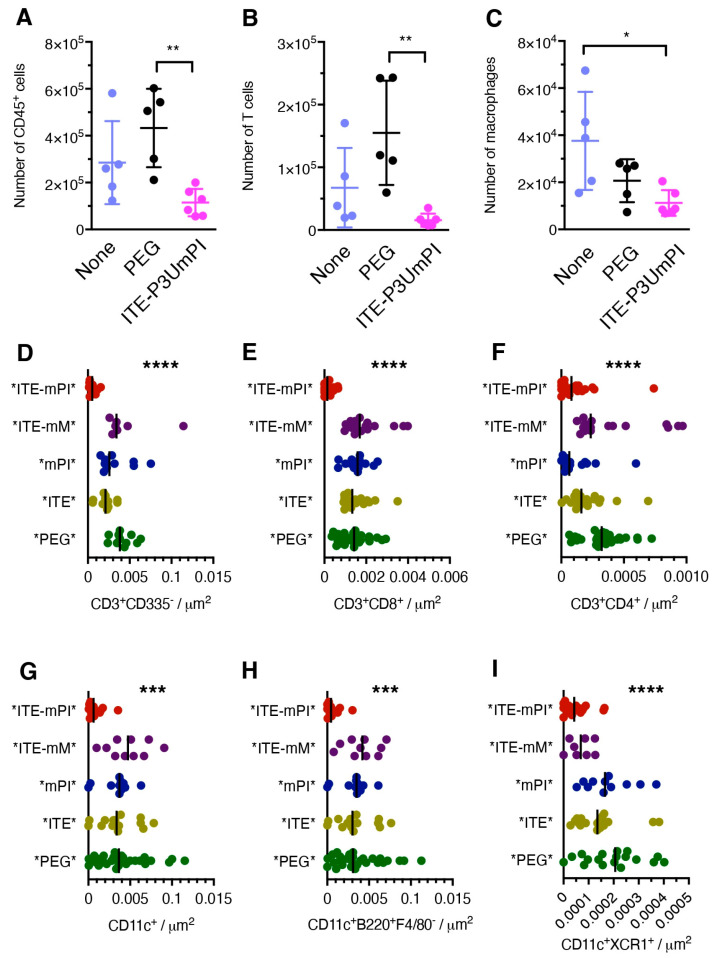
Short-term effects of NP treatment on immune cells in pancreatic islets. (**A**–**C**) Prediabetic (aged 10–12 weeks) female NOD mice (five or six per group) were treated with three injections of control NPs (“None”, “PEG”) or NPs loaded with P3UmPI + ITE during ten days before analysis of islet infiltrating cells by flow cytometry. Panels (**A**–**C**) show the total numbers obtained from islets of individual mice of CD45^+^ cells (**A**), T cells (TCR-b) (**B**), and macrophages (TCR-b^−^, CD19^−^, F4/80^+^, CD11b^lo^) (**C**). Panels (**D**–**I**) show the results of a quantitative evaluation of pancreatic sections of mice (five per group) treated with the NPs indicated, using fluorescence immunohistochemistry. Immune cells were counted blindly in all islets in two sections per mouse; each dot corresponds to one islet. The panels show the number of CD3^+^ T cells (**D**), CD8^+^ (**E**) and CD4^+^ (**F**) T cells, DCs (**G**), plasmacytoid DCs (**H**), and cDC1s (**I**). Individual values, means, and standard errors (**A**–**C** only) are shown. Data were evaluated for statistical significance using two-way Anova (**A**–**C**) or one-way Anova (**D**–**I**). *, *p* < 0.05; **, *p* < 0.01; ***, *p* < 0.001; ****, *p* < 0.0001.

To identify the immune cell populations accounting for the reduced islet infiltrate, we analyzed islets from mice subjected to short-term NP treatment. Islets from mice treated with ITE-P3UmPI NPs displayed a lower total number of immune cells as well as of T lymphocytes and macrophages than islets from untreated mice or mice injected with “empty” NPs, as determined by flow cytometry (Figure 5A–C). To determine the effect of treatment with *ITE-mPI* NPs on the nature and amplitude of islet infiltration, we undertook a more exhaustive examination of pancreatic sections by fluorescent immunohistochemistry, using seven panels of three antibodies. These were designed to identify myeloid cell populations (macrophages, type 1 and type 2 conventional DCs, plasmacytoid DCs, neutrophils) and lymphoid populations (CD4 and CD8 T cells, B cells, plasma cells, regulatory T and B cells, NK and NK-T cells). To obtain quantitative information, cells were counted in all islets present in two pancreatic sections per mouse, with five mice per experimental group. As illustrated in Figure 5D–I, this analysis revealed a strong reduction in the total number of T cells and of the CD8^+^ and CD4^+^ subsets, as well as of DCs, including plasmacytoid DCs and cDC1s, in islets from mice treated with *ITE-mPI* NPs. The effect was particularly dramatic for CD8^+^ T cells, which were almost eliminated from islets (Figure 5D). Thus, short-term treatment with *ITE-mPI* NPs depleted key lymphoid and myeloid cells from pancreatic islets, while all control NPs, including *ITE-mMOG*, had no such effect.

### 3.6. Splenic and PLN Immune Populations in Mice Cured upon NP Treatment

**Figure 6:**
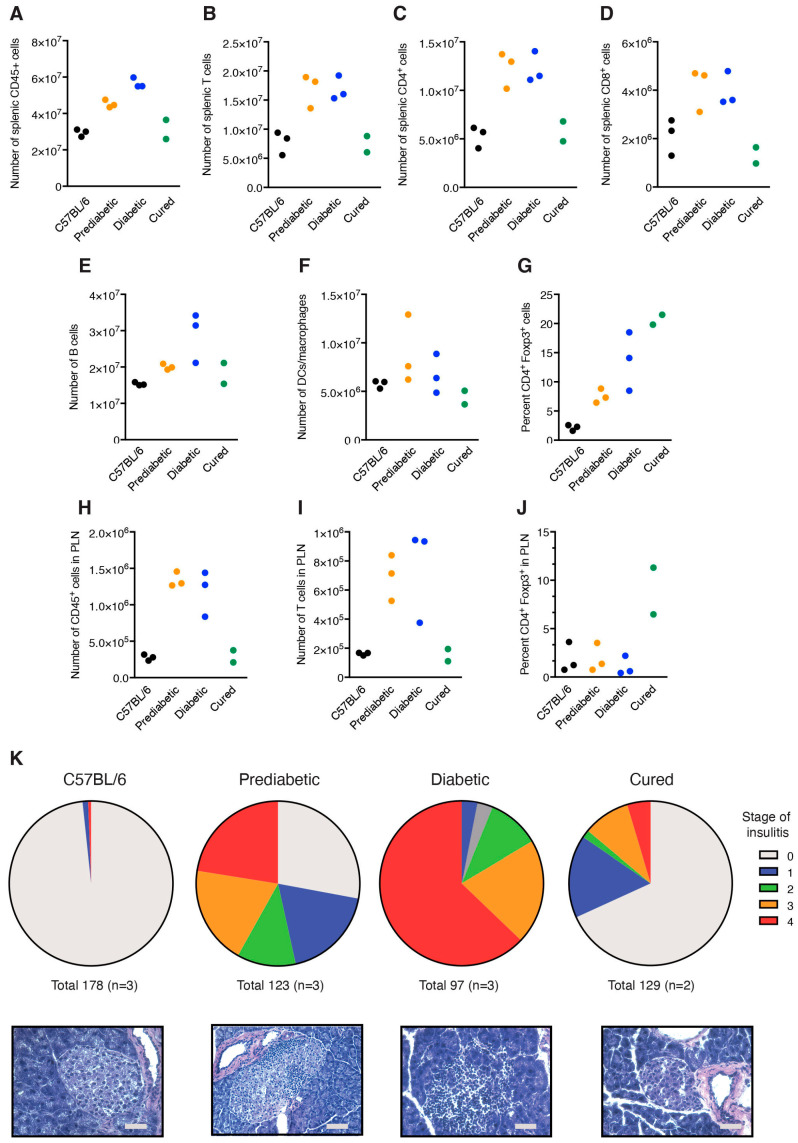
Characterization of mice in durable remission. All data are for two mice treated by ITE-P3UmPI NPs and in remission for >300 days, in comparison to C57BL/6 mice and to untreated pre-diabetic (12-week-old) and diabetic NOD mice (N = 3 for each group). Panels (**A**–**J**) represent data obtained using flow cytometry. Panels (**A**–**G**) show the total numbers of the indicated cell populations in the spleens and the percentage of Foxp3 expression among CD4^+^ cells in the four groups, while the data in panels (**H**–**J**) represent cells from PLNs. In (**K**), insulitis was scored in a blinded manner for islets from mice of the four groups. The number of islets and mice is shown below the graphs. n = 2 (cured mice) or 3 (other groups).

Although only two mice subjected to treatment with ITE-P3UmPI NPs underwent a durable remission (>300 days after treatment), we were interested in examining the immune cells in these mice, wondering how they related to short-term-treated mice. We compared splenic and PLN immune cell populations in these mice to the equivalent populations in non-autoimmune C57BL/6 and in pre-diabetic and overtly diabetic NOD mice using flow cytometry. While the spleens of untreated NOD mice contained greater numbers of CD45^+^, TCR-b^+^, CD4^+,^ and CD8^+^ T cells than the spleens of control C57BL/6 mice, the size of these populations was identical to that of non-autoimmune mice in the spleens of the “cured” animals (Figure 6A–D). Remarkably, the reduced cellularity extended to splenic B cells and DCs/macrophages, two populations found to be increased upon short-term treatment (Figure 6E,F). Conversely, the percentage of Foxp3^+^ regulatory T cells—a population not amplified upon short-term treatment—among CD4^+^ T cells was strongly increased in the spleens of “cured” mice (Figure 6G). The findings for splenocytes were mirrored for PLN cells, with normalized CD45^+^ and T cell numbers, and a strongly increased percentage of CD4^+^Foxp3^+^ cells (Figure 6H–J).

Interestingly, the numerical reduction in T cell numbers was associated with a dramatic conversion of splenic and PLN CD4^+^ and CD8^+^ T to memory cells (Supplementary Figure S6A–H). Thus, while the spleens of C57BL/6 and untreated NOD mice contained about equal numbers of naïve and memory CD4^+^ T cells, the spleens and PLN of cured mice harbored almost exclusively memory cells (Supplementary Figure S6A). Moreover, about 30 percent of splenic and PLN CD4^+^ and CD8^+^ T cells from “cured” mice produced IFN-γ—a percentage not, or rarely, found in C57BL/6 or untreated NOD mice (Supplementary Figure S6E–H). Histological analysis of islet inflammation was consistent with the reduced cellularity and the presence of Tregs in spleen and PLNs. Thus, while >90% of islets from diabetic mice were infiltrated at stage 2 or higher, two-thirds of islets from “cured” mice were devoid of inflammation, and half of the remaining islets showed low-grade stage 1 infiltration (Figure 6K). Thus, although the extent of islet infiltration in “cured” mice resembled that obtained upon short-term treatment with *ITE-mPI* NPs, examination of the spleens and PLN of “cured” mice revealed an immune state different from that observed in short-term-treated mice. Importantly, this was associated with an increased proportion of regulatory T cells among CD4^+^ cells and a massive conversion of T lymphocytes to memory cells frequently producing IFN-g, effects never observed upon short-term treatment.

## 4. Discussion

Biological as well as manufactured micro- and nano-particles have previously been shown to be of interest for the prevention and/or treatment of various autoimmune conditions [12,18,19]. Here, we report the finding that NPs can be formulated such that their principal “therapeutic” effect is a dramatic depletion of infiltrating lymphoid and myeloid cells from pancreatic islets associated with remission of disease. Importantly, in most mice overall and in all mice treated with NPs loaded with PI produced in insect cells, remission was temporary. Therefore, although the strong effect on islet infiltration suggests a translational potential of our NPs, they are unlikely to be of interest as a stand-alone therapy.

Considering previous reports on the effect of ITE-antigen NPs in murine models of multiple sclerosis and T1D [27,28], we expected to see tolerogenic effects on DCs with a resulting reduction in Th1 and Th17 T cells and an increase in Foxp3^+^ Treg and/or IL-10-producing Tr1 cells. However, although PLN DCs displayed reduced CD86 expression, a hallmark of tolerogenic DCs [34], this was not the case for splenic and islet-infiltrating DCs and macrophages after a 10-day treatment with NPs. We also did not observe an increase in the numbers of Tregs and Tr1 cells upon short-term NP treatment, although the proportion of Foxp3^+^ CD4^+^ was substantially increased in mice with durable remission. Thus, although we cannot rule out that more significant effects matching previous reports [27,28] were present at other time points, our observations provide only limited support for a key role of direct and immediate NP effects on DCs and T cells in their therapeutic efficacy.

One of the key differences from previous reports was the enhancing effect of NPs carrying fusion proteins on the numbers of splenic and PLN B lymphocytes and myeloid cells, suggesting that these cells proliferated, although migration could also explain this phenomenon. As this effect was observed for both PI and the control protein, it was likely due to the common moieties of the fusion proteins and/or contaminants. Since ubiquitin is a highly conserved and abundant self-protein, human ubiquitin is unlikely to be immunogenic in mice. In contrast, protein G is expressed by streptococci of the C and G serotypes, which can be found as commensals or pathogens in humans and many animals [35] and may therefore have given rise to specific antibody production by plasma and B cells. Moreover, our fusion proteins bind efficiently to murine IgG [23] so that fusion protein-loaded NPs may have formed immune complexes upon i.v. injection. Immunoglobulins are known to be adsorbed in serum to the “biomolecular corona” of many NPs and thought to enhance NP uptake through Fc receptors [36,37]. As immunoglobulin decoration of our NPs is expected to exceed the non-specific adsorption to other NPs, our NPs may be internalized very efficiently by Fc-γ receptors expressed by myeloid and possibly also B cells. B cells express exclusively the inhibitory FcγRIIB receptor, which is also dominant on DCs and present among other FcRs on macrophages [38,39]. However, a comparison with NPs carrying PI without the protein G moieties will be required to conclude on a potential role of these moieties and their interaction with Fc receptors. Both FcγRIIB signaling and ITE effects can explain CD86 downregulation on DCs and macrophages, while CD86 upregulation on B cells cannot be related to FcγR signaling. It is possible that the strong B cell response to P3UmPI is in part due to a pre-existing repertoire of insulin-specific B cells in NOD mice, explaining splenocyte and B cell amplification in this strain but not in C57BL/6 mice (Figure 2A,B).

The second payload of our NPs, the AhR ligand ITE, had several biological effects, not all of which are readily explained by the documented actions of AhR agonists [40]. NPs loaded with ITE alone delayed diabetes progression moderately, a plausible result given the known effects of ITE on DCs and T lymphocytes. ITE loading was also a condition for the suppressive effect of *mPI* NPs on insulitis, which may be related to the same effects. However, it is less clear how AhR signaling promotes the effects of the fusion proteins in PLN, i.e., increases in the number of DCs and macrophages as well as plasma cells and marginal zone-like B cells (Figure 3).

We were surprised to observe that NP treatment induced secretion of IL-10 and other cytokines, including TGF-β and IL-4, by purified splenic B lymphocytes. As the effect was somewhat more pronounced for NPs loaded with bacterially expressed protein, we suspected LPS contamination as the underlying reason. However, comparison with LPS standards suggested that LPS alone was unlikely to account for the effect. As our fusion proteins were produced using a single step of affinity purification, a significant amount of unidentified contaminant proteins was not eliminated (Supplementary Figure S1B,C). Such contaminants may have contributed to stimulating IL-10 expression by splenic B cells from both C57BL/6 and NOD mice in vitro (Supplementary Figure S5B). However, B cell expression of TGF-β in vitro (Supplementary Figure S4C) as well as amplification and activation of splenic and LN B cells in vivo (Figure 3A–E and Figure 4A) upon treatment of NOD but not C57BL/6 B cells or mice, respectively, suggests a response linked to an autoreactive B cell repertoire in NOD mice. The protective effect of adoptively transferred splenic B cells from mice treated with PI-loaded NPs (Supplementary Figure S5D) suggests that B cells producing immunoregulatory cytokines could have contributed to remission in NP-treated NOD mice.

The possibly most surprising result in this study was the strong suppression of insulitis with the near-disappearance of T cells and especially CD8^+^ T lymphocytes and myeloid cells, including cDC1, known to play a key role in priming islet autoimmunity [41]. While further studies will be required to reveal the underlying mechanism, we propose the following, admittedly highly speculative, scenario. Given the demonstration that our NPs accumulate in the pancreas [22] and are efficiently taken up by DCs, many islet-infiltrating myeloid cells may (cross-) present insulin to T cells. Since many CD8^+^ T cells in islets recognize PI [42] and since islet CD8^+^ T cells have high cytotoxic potential [43], strong up-regulation of PI cross-presentation may result in the killing of myeloid cells by CD8^+^ T cells primed in PLNs. Given the effect of ITE on myeloid cells, killer T cells may receive insufficient costimulatory signals for survival [44]. Direct effects of ITE on T cells may also contribute to reduced survival. Alternatively, T cells may have migrated to bulk organs such as the liver and lungs, where cross-presentation of PI will also occur. Note that the percentage of CD8^+^ T cells was also reduced in spleens (Figure 2J).

It is also interesting to speculate how NP treatment may have achieved indefinite remission in two mice. Histological examination of islets from the “cured” mice revealed a state equivalent to mice subjected to short-term treatment with *ITE-mPI* NPs. In contrast, analysis of their spleens and PLN cells revealed a state not achieved in short-term treatments, i.e., a global reduction in immune cell subsets, including B cells, to reach the levels of a non-autoimmune mouse, except for Tregs, which were far more abundant than in the latter. Thus, in these mice, B cell and myeloid cell responses in spleens and PLNs may have evolved in a manner allowing them to develop efficient regulation by CD4^+^ T cells and to maintain the suppression of insulitis. However, we do not know whether and how this state is linked to the short-term effects of NP treatment, including IL-10 production by B cells. It cannot be ruled out that the stimulation of IL-10-producing B lymphocytes by ITE-P3UmPI NPs was instrumental in this outcome; however, this remains speculative in the absence of experimental evidence. Induction of Tr1 and Foxp3^+^ Tregs by B cells producing IL-10 is a well-described phenomenon [45,46,47,48]. We also noted a striking increase in memory CD4^+^ and CD8^+^ T cells producing IFN-γ in the spleens and PLN of the cured mice. We do not know whether these cells contributed to controlling autoimmunity. However, cells with the same phenotype, killing cells presenting beta cell antigens, have been described by Santamaria and colleagues in NOD mice undergoing T1D remission upon treatment with NPs carrying recombinant peptide-MHC complexes [49,50].

## 5. Conclusions

Our results indicate that NPs loaded with PI fusion proteins and ITE can counteract autoimmune diabetes in the NOD model through two mechanisms: (at least temporary) attenuation of islet infiltration by CD8^+^ T cells and DCs, and induction of B lymphocytes producing regulatory cytokines. The exact mechanisms underlying both phenomena, as well as their role in disease remission, remain to be determined through future research. However, it is unlikely that the NPs can be sufficient as a stand-alone therapy to induce durable remission in a satisfactory proportion of mice, and by extension, patients. We speculate that the strong suppressive effect of the NPs on the insulitis may be of interest in combination with strategies inhibiting the reentry of immune cells in islets and generating or enhancing regulatory T cells. This might be achievable by accelerating the process that is likely to have occurred in the spleens and PLNs of our “cured” mice, by methods to promote the expansion and function of endogenous regulatory T cells, or by cell therapies with bulk or antigen-specific regulatory cells.

## Data Availability

The raw data supporting the conclusions of this article will be made available by the authors on request.

## Supplementary Materials

The following supporting information can be downloaded at: https://www.mdpi.com/article/10.3390/cells15020174/s1, Table S1: Antibodies used in flow cytometry experiments with P3UmPI-loaded NPs; Table S2: Antibodies used in flow cytometry experiments with *mPI* and *mMOG* loaded NPs; Table S3: Panels used for immunofluorescence histology; Figure S1: Production of insect cell-expressed fusion proteins; Figure S2: Gating strategies for analysis of myeloid and lymphoid splenic and PLN cells by flow cytometry. Figure S3: Effect of short-term in vivo treatment with vehicle, PEG-coated or ITE-P3UmPI-loaded NPs on function, phenotype and numbers of splenocytes; Figure S4: Analysis of IL-10 secretion by B cells in response to NP stimulation; Figure S5: Effect of splenocyte incubation ex vivo with NPs on proliferation and cytokine secretion; Figure S6: Memory phenotype and IFN-γ production by T cells in mice with durable remission.

## Abbreviations

The following abbreviations are used in this manuscript:

AhR	Aryl hydrocarbon receptor
BM-DC	Bone marrow-derived dendritic cells
DCs	Dendritic cells
ITE	2-(1′H-indole-3′-carbonyl)-thiazole-4-carboxylic acid methylester (ITE)
LN	Lymph node
LPS	Lipopolysaccharide
mMOG	Murine myelin oligodendrocyte protein
mPI	Murine proinsulin
NOD	Non-obese diabetic (mouse)
NPs	Nanoparticles
PLN	Pancreatic lymph node
PI	proinsulin
T1D	Type 1 diabetes
