# Supplementary material for: Proinsulin-Loaded Nanoparticles Suppress Insulitis and Induce Temporary Diabetes Remission
Source: Cells. 2026 Jan 19;15(2):174. doi: 10.3390/cells15020174 (PMC12839391; doi:10.3390/cells15020174)
Supplement: Supplementary file 1 [file cells-15-00174-s001.zip › cells-4074145-supplementary.pdf]

Supplementary Material

Detailed Methods

Fusion protein expression in E. coli and purification. Expression of the fusion cassette, cloned in pET16b, by BL21DE3 bacteria was induced during 4 hours by Isopropyl-β-D-thiogalactoside from Sigma-Aldrich (Saint Louis, MO, USA). Then bacteria were pelleted, lysed 30 min on ice in a lysis buffer (Tris 50 mM, NaCl 50 mM, TCEP 1 mM, EDTA 0.5 mM, glycerol 5%, pH=8), lysozyme 0.2 mg/mL and DNase I 0.1 mg/mL. After addition of Triton 1% for 15 min and centrifugation at 20,000x*g* for 1 h at 4°C, the supernatant was passed over a rabbit IgG-Sepharose column. P3UmPI was eluted with a CHAPS 1%/CAPS 20mM buffer, is dialyzed overnight (MWCO 8000 kDa) in PBS glycerol 10% and cleaned up by passing through columns for removal of detergent (Pierce™ Detergent Removal Spin Column; Thermofisher Scientific, Waltham, Massachusetts) and endotoxin (Endotoxin Removal Spin Column; Thermofisher). P3UmPI concentration was measured using Qubit equipment (Thermofisher).

Insect cell culture. Sf9 (*Spodoptera frugiperda*) and Hi5 (*Trichoplusa ni.*) cells were cultured in 20mL serum free medium (Sf-900™ III SFM - Gibco™ - 12658019 and Express Five™ SFM - Gibco™ – 10486025 respectively) in 175cm^2 Nunc™ EasYFlask™ flasks (ThermoFisher – 159910) at 27°C. Flasks were split upon reaching 80% confluency, cells were detached by mechanical disruption of the monolayer and diluted 5-fold. For transfection and infection experiments, cells were incubated at 27°C for 3 hours after seeding to form an attached monolayer before conducting the experiment.

Plaque Assay. Triplicates of 1.2x10^6 Sf9 cells seeded in each well of a 6 well plate (Nunc) were infected for 2 hours at 27°C in a humid chamber with 300µL of serial dilutions from 10^{-2} to 10^{-5} of baculovirus containing supernatant prepared in serum free culture medium, with 2 wells exempt of virus, kept as a negative control. After infection, cells were covered with 1,5% low-melting agarose in Grace's Insect Medium 1X (Gibco) supplemented with 5% FCS (Eurobio), after solidification of the agarose overlay, 1mL of Grace's Insect Medium 1X (Gibco) supplemented with 5% FCS was added on top of the agarose. After 5-7 days incubation at 27°C in a humid chamber, Grace's Insect Medium was discarded, and cells stained with 1mL Neutral Red 0,033% (Sigma) for 2 hours. Plates were flipped on top of absorbent paper to remove excess staining and lysis plaques were defined as colourless spots. 12 recombinant clones were harvested by lightly scratching each lysis plaque with a 1000uL pipette and subsequently incubated with 15000 Sf9 cells in a 96 well plate (Nunc). After 3-5 days

incubation, supernatant was harvested and stored at 4°C for future Sf9 infection and production of high titer viral stocks.

Insect cell infection to produce high titer viral supernatant and fusion proteins: $1.5x10^6$ Sf9 cells were infected with 30µL of viral supernatant in 600µL of culture medium in $25cm^2$ Nunc™ EasYFlask™ flasks (ThermoFisher) for 1 hour at 27°C. Viral supernatant was replaced with 5mL of culture medium, after 5 days incubation at 27°C, supernatant (called SN5) was harvested, dead cells were removed by spinning at 1200rpm for 5 minutes and SN5 was stored at 4°C. $20x10^6$ Sf9 cells were infected with 150µL of SN5 in 3mL of culture medium in $175cm^2$ Nunc™ EasYFlask™ flasks for 1 hour at 27°C. Viral supernatant was replaced with 20mL of culture medium, after 5 days incubation at 27°C, supernatant (called SN20) was harvested, dead cells were removed by spinning at 1200rpm for 5 minutes and SN20 was stored at 4°C. This step was repeated with SN20 as infectious reagent in order to restock SN20.

To produce fusion proteins, $60x10^6$ Hi5 cells were infected with 5mL of SN20 in $175cm^2$ flasks for 1 hour at 27°C. Viral supernatant was replaced with 25mL of culture medium, after 5 days incubation at 27°C, supernatant (SNp) was harvested, dead cells were removed by spinning at 1200rpm for 5 minutes, SNp was filtered at 0.22µm (Millipore) and stored at 4°C.

Purification of proteins from insect cell supernatants. For *mPI* purification, 200-250mL of SNp were passed over 5mL of rabbit Iimunoglobulin-coupled Sepharose 4B. For *mMOG* purification, 10mL of SNp were passed over 3mL of rabbit Immunoglobulin-coupled sepharose for 4 hours at 4°C. Bound protein was eluted using a buffer containing 20 mM N-cyclohexyl-3-aminopropanesulfonic acid (pH 12) and 1% 3-[(3-cholamidopropyl) dimethylammonio]-1-propane sulfonate. Eluted fractions containing the fusion proteins were neutralized, pooled, dialyzed against PBS, and stored in aliquots at −80°C. Standard denaturing SDS-PAGE analysis was performed using NuPAGE™ (Invitrogen) equipment. For each quantification, BSA standards were prepared. After overnight Coomassie blue staining (0,006% coomassie in 10% acetic acid), pictures were taken with ChemiDoc (Bio-Rad). Protein concentration was estimated against the BSA standard curve and estimations were corrected to account for the differential binding of Coomassie to proteins (41).

Loading of USPIO-PO-PEG-COOH NPs. 9nm non-coated NPs were synthesized by the reaction of Iron (III) acetyl acetonate with benzylalcohol at 250°C during 30 min under microwave irradiation, as described (22). After magnetic separation and washing, the NPs were coated with PO-PEG-COOH and finally dispersed in water at pH=7.4. NPs were first loaded with ITE in DMSO, which was added to USPIO-PO-PEG-COOH NPs in water at room temperature with a ratio $R_{ITE/NP}$ = 600. After 2 h under mixing, P3UmPI, *mPI* or

mMOG were coupled to NPs in coupling buffer (Pluronic ® F-127 (Sigma) 3g.L^{-1}, H_3PO_4 (Sigma) 0.5µmol.L^{-1}, pH=6) in a two-step procedure (activation and conjugation) at 37 °C. First, the carboxylic acid functions at the outer surface of the NPs were activated using 1-ethyl-3-(3-dimethylaminopropyl) carbodiimide (EDC, Alfa Aesar, $n_{EDC}= 5n_{COOH}$) at pH = 6 during 10 min. The second step was the linkage of the amine function of the protein with the activated carboxylic acid functions on the NPs. The protein was added with the ratio R = n_{*mPI*}/n_{NP}=8 at pH=6 to the ferrofluid during 1 h at 37 °C. The NPs were washed in a 5% glucose solution by ultracentrifugation three times for 15 min (Amicon 100 kDa, Merck Millipore) and stored in 100uL aliquots at -20°C. At various points during the coupling the pH was measured and adjusted to 7.4 using NaOH (10^{-1} mol.L^{-1}) solution. For physicochemical characterizations, the NPs were redispersed in milliQ water.

Physicochemical NP characterization : Iron concentration was estimated through destructive dosage of iron by heating 10µL of a 4 fold dilution of the NP into 100µL 7N nitric oxide and 100µL of 20% hydrogen peroxide overnight. The absorbance at 477 nm was recorded after addition of 1mL milliQ water and 100uL 2M potassium thiocyanate. Molar concentrations were estimated with the following equations : $C_{Fe} = A_{477}/7322$ and $CNP_{9,5}$ = $C_{Fe}/16500$. The average number of ITE per NP was evaluated using UV–vis spectroscopy. ITE saponification was performed by adding 975µL NaOH 1 mol.L^{-1} to ITE or ITE-loaded NP for 2h (25µL of NP). NPs were isolated from supernatant using magnetic decantation. The resulting carboxylate ion (carboxylate ITE) was water soluble and characterized by two UV bands at 279 and 388 nm. A calibration curve was established after basic hydrolysis of ITE alone and the average number ITE per nanoparticle was deduced from this curve. The conjugation efficiency of the fusion protein *mPI* or *mMOG* was investigated using the same SDS-PAGE method as described for protein purification. Complete NPs were determined to contain 348 ± 70 molecules ITE and 4.3 molecules of fusion proteins (P3UmPI, *mPI*, *mMOG*) per particle.

Supplementary Table S1. Antibodies used in flow cytometry experiments with P3UmPI-loaded NPs

Specificity	Clone	Manufacturer
CD16/CD32	93	Biolegend
CD45	30-F11	eBioscience
CD11b	M1/70	eBioscience
F4/80	BM8	eBioscience
CD11c	N418	eBioscience
TCRb	H57-597	eBioscience
CD19	1D3	eBioscience
CD206	C068C2	Biolegend
CD5	53-7.3	Miltenyi
CD1d	K253	Biolegend
CD21	7 E9	Biolegend
B220	RA3-6B2	Biolegend
CD138	281-2	Biolegend
CD86	GL-1	Biolegend
CD8a	53-6.7	eBioscience
CD4	RM4-5	Biolegend
CD23	B3B4	Biolegend
CD44	IM7	eBioscience
CD62L	MEL-14	Biolegend
LAP	TW7-16B4	eBioscience
IL-4	11B11	eBioscience
IL-10	JES5-16E3	eBioscience
IDO	2E2/IDO1	Biolegend
Foxp3	FJK-16s	eBioscience

Supplementary Table S2. Antibodies used in flow cytometry experiments with *mPI* and *mMOG* loaded NPs

Marker	Clone	Fluorochrome	Provider	Reference	Dilution
**Myeloid panel**					
CD45	30-F11	BV786	BD Horizon	564225	1/300
TCRb	H57-597	Biotine	BioLegend	109203	1/100
CD19	eBio1D3	APCeF780	Invitrogen	47-0193-82	1/100
CD49b	DX5	Biotine	eBiosciences	13-5971-82	1/100
Ly6G	RB6-8C5	BV510	BioLegend	108438	1/100
F4/80	BM8	PECy7	BioLegend	123113	1/200
B220	RA3-6B2	SB600	Invitrogen	63-0452-80	1/100
MHC-II (I-A^{g7})	10-3.6	PE	BioLegend	109908	1/500
CD11c	N418	BV711	BioLegend	117349	1/100
CD11b	M1/70	AF488	BioLegend	101219	1/100
CD172	P84	PerCPCy5.5	BioLegend	144010	1/200
XCR1	ZET	AF647	BioLegend	148214	1/200
Secondary reagent					
Streptavidine	n/a	APCeF780	eBioscience	47-4317-82	1/200
**Lymphoid panel**					
Surface labeling					
CD45	30-F11	PerCPCy5.5	BioLegend	103132	1/100
TCRb	H57-597	AF700	BioLegend	109224	1/50
CD4	RM4-5	PECy5	BioLegend	100513	1/100
CD8a	53-6.7	BV605	BD Horizon	563152	1/100
CD19	eBio1D3	APCeF780	Invitrogen	47-0193-82	1/100
CD21	7E9	PECy7	BioLegend	123419	1/100
CD23	B3B4	FITC	Invitrogen	11-0232-81	1/100
CD138	281-2	BV421	BioLegend	142523	1/2000
TACI	eBio8F10-3	PE	Invitrogen	12-5942-81	1/100
CD62L	MEL-14	BV785	BioLegend	104440	1/100
Nuclear labeling					
Foxp3	FJK-16s	APC	Invitrogen	17-5773-82	1/20

Supplementary Table S3. Panels used for fluorescent immunohistochemistry

Antigen	Clone	Conjugation	Provider	Reference	Dilution	Secondary	Provider	Ref.	Dilution
**Macrophage, dendritic cells, plasmacytoid dendritic cell, B lymphocytes**									
Nucleus		DAPI	Roche	10236276001	1/5000	n/a	n/a	n/a	n/a
B220	RA3-6B2	Biotine	BD Pharm	553086	1/300	AF647-Strepta	Invitrogen	S32357	1/1000
F4/80	BM8	AF488	Biolegend	123120	1/25	n/a	n/a	n/a	n/a
CD11c	N418	Pur Hams	Biolegend	117302	1/25	AF594-GâH	Invitrogen	A78961	1/1000
**Regulatory T lymphocytes**									
Nucleus		DAPI	Roche	10236276001	1/5000	n/a	n/a	n/a	n/a
CD4	GK1.5	AF488	Biolegend	100423	1/50	n/a	n/a	n/a	n/a
FOXP3	FJK-16s	Biotine	invitrogen	13-5773-82	1/100	AF647-Strepta	Invitrogen	S32357	1/1000
**Regulatory B lymphocytes**									
Nucleus		DAPI	Roche	10236276001	1/5000	n/a	n/a	n/a	n/a
CD19	6D5	AF488	Biolegend	115521	1/25	n/a	n/a	n/a	n/a
IL10	JES5-16E3	AF647	Biolegend	505014	1/100	n/a	n/a	n/a	n/a
CD138	281-2	Bio	BioLegend	142512	1/100	AF594-Strepta	Invitrogen	S32356	1/1000
**NK cells, NK-T cells, T lymphocytes**									
Nucleus		DAPI	Roche	10236276001	1/5000	n/a	n/a	n/a	n/a
CD3e	E4T1B	Pur Rb	CST	78588	1/200	AF594-DkâRb	Invitrogen	A21207	1/1000
CD335	29A1.4	FITC	eBiosc	11-3351-82	1/10	n/a	n/a	n/a	n/a

Granzyme B	16G6	Bio	eBiosc	13-8822-82	1/100	AF647-Strepta	Invitrogen	S32357	1/1000
**Macrophages,neutrophils**									
Nucleus		DAPI	Roche	10236276001	1/5000	n/a	n/a	n/a	n/a
CD11b	M1/70	AF488	Biolegend	101219	1/25	n/a	n/a	n/a	n/a
CD169	3D6.112	AF647	BioLegend	142407	1/100	n/a	n/a	n/a	n/a
Ly6G	1A8	AF594	BioLegend	127636	1/500	n/a	n/a	n/a	n/a
**Type 1 and type 2 conventional dendritic cells**									
Nucleus		DAPI	Roche	10236276001	1/5000	n/a	n/a	n/a	n/a
XCR1	ZET	AF647	Biolegend	148214	1/25	n/a	n/a	n/a	n/a
CD172	P84	AF488	Biolegend	144024	1/25	n/a	n/a	n/a	n/a
CD11c	N418	Pur Hams	Biolegend	117302	1/25	AF594-GaH	Invitrogen	A78961	1/1000
**Helper and killer T lymphocytes**									
Nucleus		DAPI	Roche	10236276001	1/5000	n/a	n/a	n/a	n/a
CD3e	E4T1B	Pur Rb	CST	78588	1/200	AF594-DkâRb	Invitrogen	A21207	1/1000
CD4	GK1.5	AF488	Biolegend	100423	1/50	n/a	n/a	n/a	n/a
CD8a	4SM15	Biotine	eBiosc	13-0808-82	1/500	AF647-Strepta	Invitrogen	S32357	1/1000

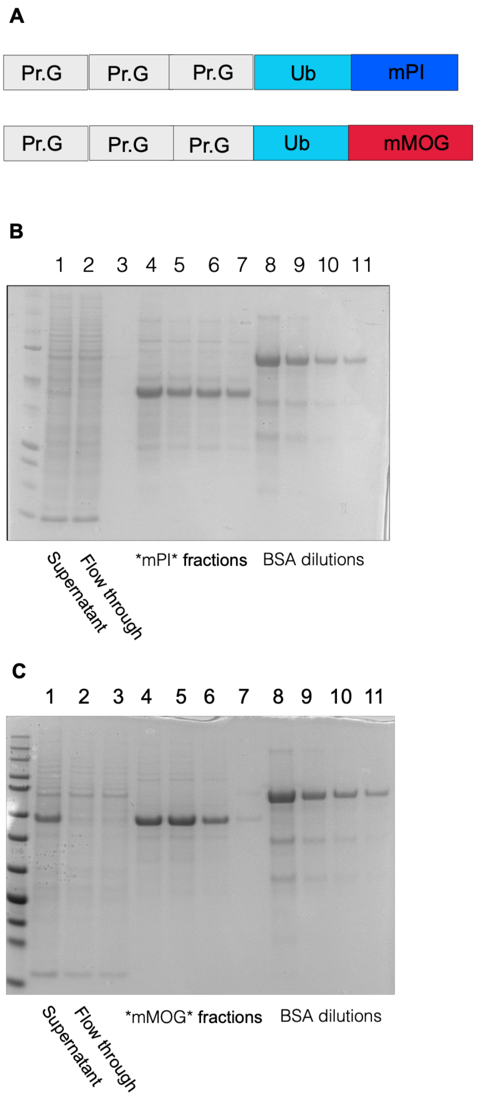

Supplementary Figure S1. **Production of insect cell-expressed fusion proteins**. Panel (**A**) shows the composition, drawn to scale, of the *mPI*/P3UmPI and *mMOG* fusion proteins. Pr.G, streptococcal Protein G (B1 domain; 54 amino acids); Ub, ubiquitin (81 amino acids); mPI, murine PI 1 (84 amino acids); mMOG, murine myeline oligodendrocyte protein fragment (residues 25-155; 131 amino acids). Panels (**B**) and (**C**) show examples of the purification of insect cell-expressed *mPI* and *mMOG* proteins; the bovine serum albumin standards correspond to serial two-fold dilutions of bovine serum albumin (BSA), starting with 4.5 μg in lanes 8.

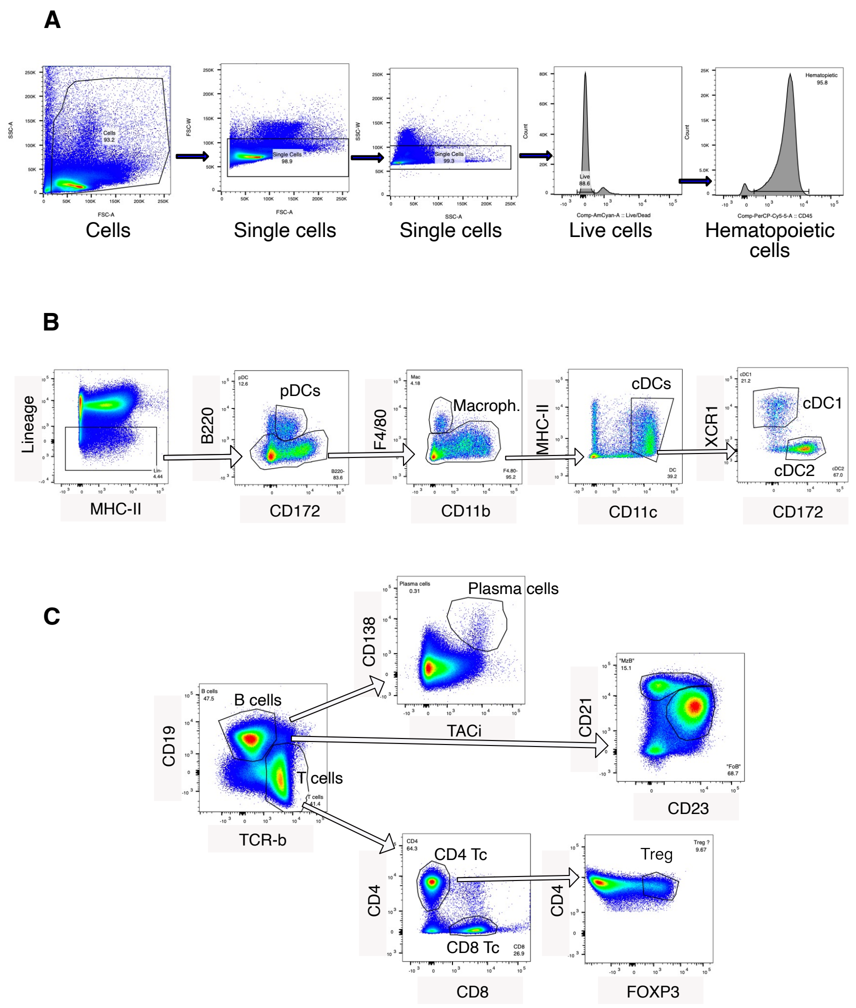

Supplementary Figure S2, related to Figures 2, 3, 5 and 6. Gating strategies for analysis of myeloid and lymphoid splenic and PLN cells by flow cytometry. Gating for both populations started with selecting single live CD45^{+} hematopoietic cells, as shown in **A**. Myeloid cells were selected based on absence of lineage markers (identifying B, T and NK cells) and identified as plasmacytoid DCs (B220^{+}, CD172^{intermediate}), macrophages (F4/80^{+}), conventional DCs (cDCs, CD11c^{+}) and cDC1s (XCR1^{+}) and cDC2 (CD172^{+}), as shown in panel **B**. Lymphoid cells were identified as B cells (CD19^{+}) subdivided in plasma cells

(CD138^{+}, TACi^{+}), marginal zone-like (CD21^{+}CD23^{-}) and follicular (CD21^{-}CD23^{+}) B cells, and T cells (TCR-β^{+}) subdivided in CD8^{+} and CD4^{+} T cells with the subpopulation of regulatory T cells (CD4^{+}Foxp3^{+}), as illustrated in panel **C**.

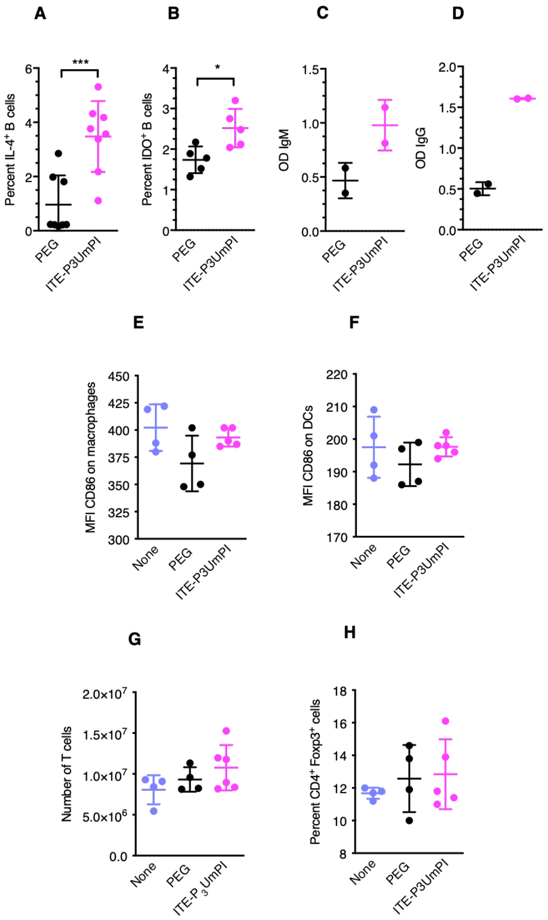

Supplementary Figure S3, related to Figure 3. **Effect of short-term *in vivo* treatment with vehicle, PEG-coated or ITE-P3UmPI-loaded NPs on function, phenotype and numbers of splenocytes.** Splenocytes from mice subjected to short-term NP treatment as in Fig. 3 A-H were examined for IL-4 (**A**) and indoleamine 2,3 dioxygenase (**B**) expression by flow cytometry. Panels (**C**) and (**D**) show the total serum concentrations of IgM (**C**) and IgG (**D**) for 2 mice of each group from the same experiment. Panels E-H show the CD86 expression levels on macrophages (**E**) and DCs (**F**) the total number of T cells (**G**) and the percentage of Foxp3^{+} cells among CD4^{+} T lymphocytes (**H**). Data were analyzed by one-way Anova (**A-D**) or two-way Anova (**E-H**). *, $p<0.05$; **, $p<0.01$; ***, $p<0.001$.

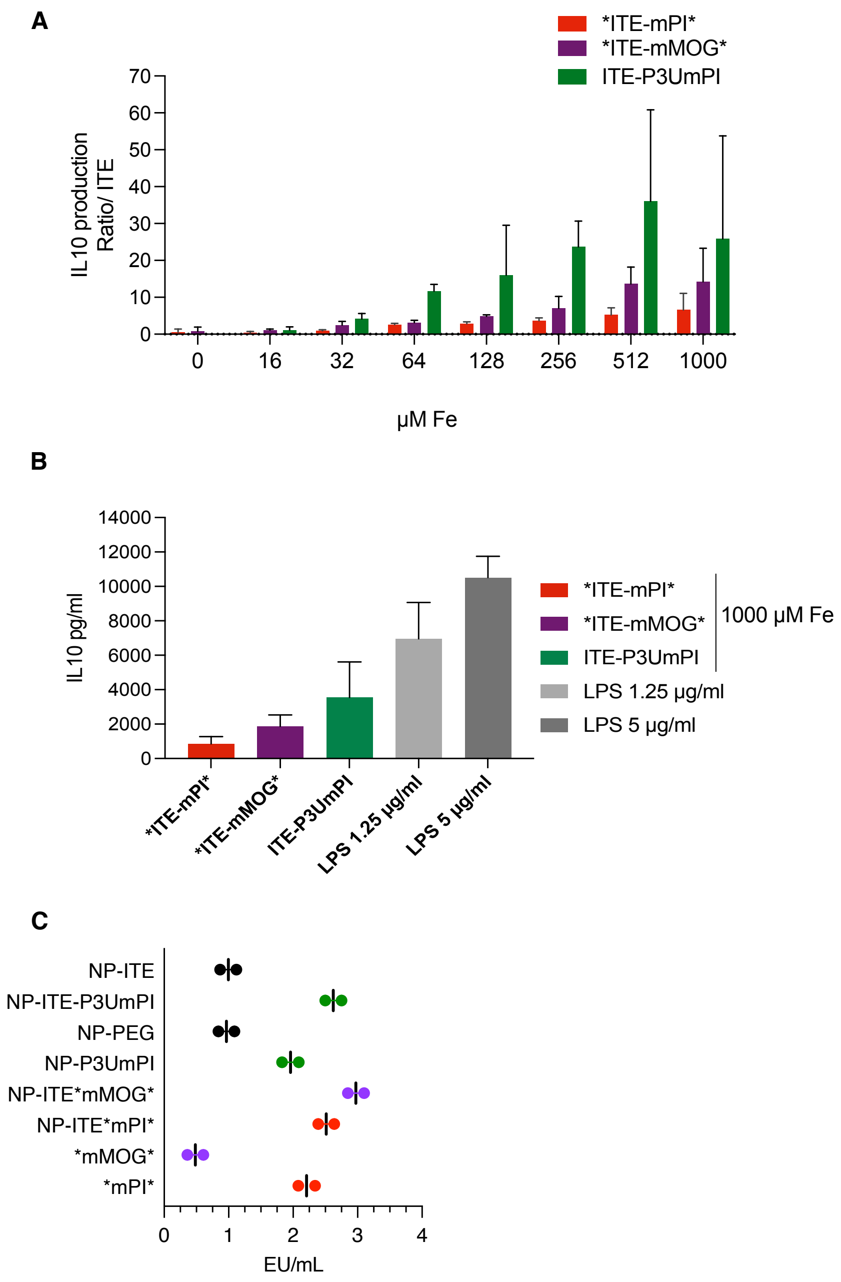

Supplementary Figure S4, related to Figure 3. **Analysis of IL-10 secretion by B cells in response to NP stimulation**. **A**) Purified splenic B cells from 10 week-old female NOD mice were subjected to stimulation with graded amounts of NPs loaded with ITE plus *mPI*, *mMOG* and P3UmPI, as indicated. IL10 secretion in supernatants (pg/ml) was measured by ELISA after 72h of culture. Data for 3 mice were normalized with respect to values for ITE-only NPs set at 1. (**B**) Comparison of IL-10 secretion by B cells stimulated with LPS at concentration of 1.25 and 5 µg/ml alongside various NP formulations at 1000 µM Fe. (**C**) Endotoxin content of fusion proteins and protein + ITE-loaded NPs was measured in duplicates by chromogenic commercial assay.

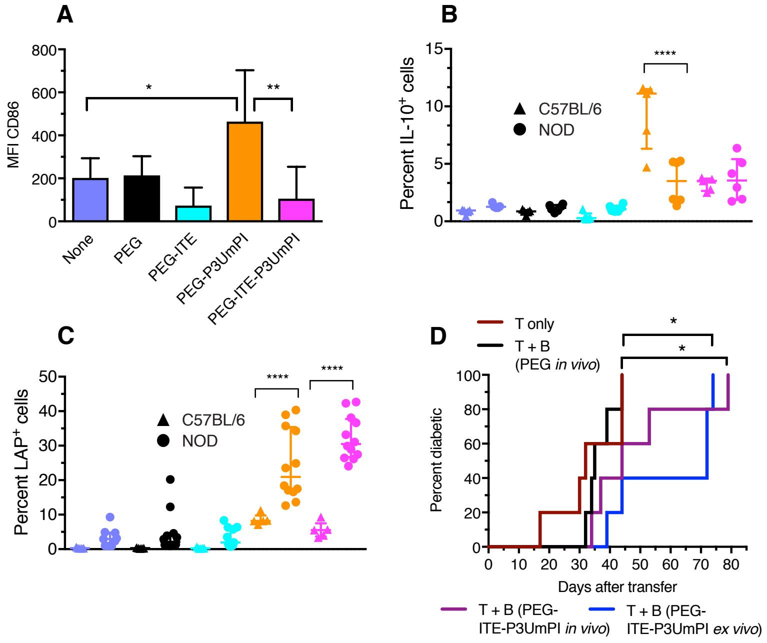

Supplementary Figure S5, related to Figure 3. **Effect of splenocyte incubation *ex vivo* with NPs on proliferation and cytokine secretion**. Splenic B cells were sorted from female NOD and C57BL/6 mice and incubated with NPs for 3 days. Panel (**A**) shows the effect of treatment on surface level of CD86 on NOD B cells. In (**B**) and (**C**), B cells from C57BL/6 (triangle) and NOD (circle) mice were compared with respect to production of IL-10 (**B**) and LAP (**C**), determined by intracellular staining. In (**D**), NOD $Rag^{-/-}$ mice (n=5 per group) were injected with sorted splenic T cells obtained from newly diabetic NOD mice alone, or together with splenic B cells from prediabetic NOD mice injected 3 times with PEG-coated NPs or with ITE-P3UmPI-loaded NPs, or together with B cells treated *ex vivo* with the latter NPs. n = 3 to 6 from 3 independent experiments. Group mean +/− SD values were compared using the two-way ANOVA test. Diabetes incidence with n=5 per group was compared with the log-rank test.

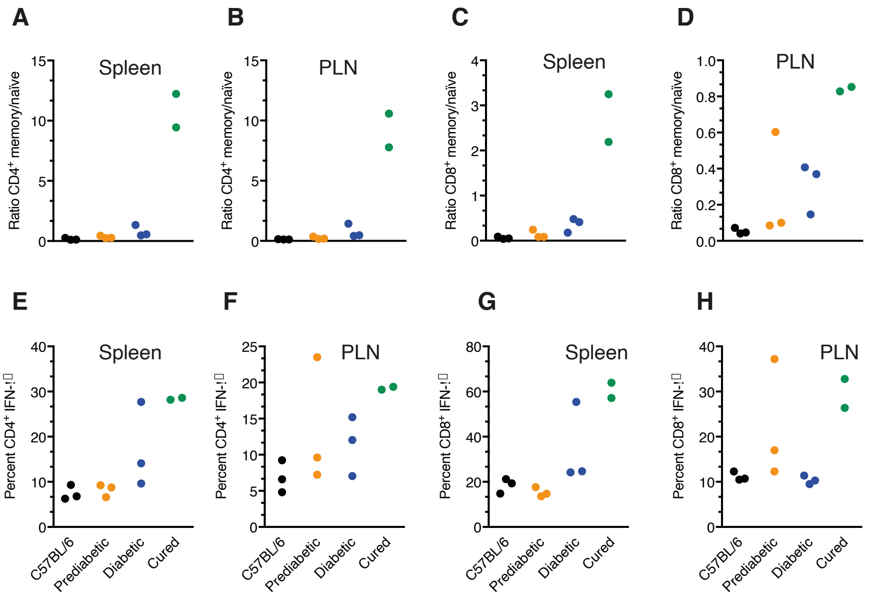

Supplementary Figure S6, related to Figure 6. **Memory phenotype and IFN-γ production by T cells in mice with durable remission.** (**A-D**) The ratio of memory (CD4^{+}CD62L-) to nave (CD44^{-}CD62L^{+}) cells was determined for splenic and pancreatic lymph node CD4^{+} and CD8^{+} T cells in control C57BL/6 mice as well as in prediabetic, diabetic and NP-cured NOD mice. Panels (**E-H**) show the percentage of IFN-γ-producing CD4^{+} and CD8^{+} T cells, as detected by intracellular cytokine staining, in the spleen and pancreatic lymph nodes of the four groups of mice. N = 2 (cured mice) or 3 (other groups).
